# Antigen‐specific polyfunctional cytotoxic T cells differentiate intraocular from peripheral blood immune responses in posterior uveitis

**DOI:** 10.1002/cti2.70036

**Published:** 2025-05-15

**Authors:** Kaiser Alam, Arun Raina, Bibhuprasad Das, Sandhya Bhanja, Sayantan Ghosh, John V Forrester, Soumyava Basu

**Affiliations:** ^1^ Ocular Immunology Laboratory, Prof Brien Holden Eye Research Centre LV Prasad Eye Institute Hyderabad India; ^2^ Multidisciplinary Unit of Research on Translational Initiatives (MURTI) Research Centre, Visvesvaraya Bhavan, Division of Microbiology, Department of Life Sciences, School of Science Gandhi Institute of Technology and Management (GITAM) Visakhapatnam India; ^3^ Institute of Genomics and Integrative Biology New Delhi India; ^4^ Neuroimmunology Lab Yale School of Medicine New Haven CT USA; ^5^ Ocular Immunology Group, Section of Infection and Immunity, Institute of Medical Sciences University of Aberdeen Aberdeen UK; ^6^ Saroja A Rao Center for Uveitis LV Prasad Eye Institute Hyderabad India

**Keywords:** antigen, CD8 T cells, flow cytometry, polyfunctional, unsupervised clustering, uveitis

## Abstract

**Objectives:**

Peripheral blood is frequently used to study the immune response in human uveitis because of the inaccessibility of ocular tissue samples. To determine whether peripheral blood immune cells accurately reflect the intraocular immune response, we compared the T‐cell profiles and antigen‐specific cytokine responses between paired vitreous and peripheral blood samples from patients with sight‐threatening posterior uveitis.

**Methods:**

We collected paired vitreous and peripheral blood mononuclear cells (PBMCs) from 24 patients with posterior uveitis. Multi‐parametric flow cytometry was employed to identify surface and intracellular cytokine markers after activation with candidate antigenic peptides [*Mycobacterium tuberculosis* (MTb) peptides and retinal autoantigens]. Data were analysed through manual gating, unsupervised clustering and dimensionality reduction (FlowSOM, FlowJo).

**Results:**

The CD8^+^/CD4^+^ ratio in a representative set of seven paired samples was higher in the vitreous than in PBMCs. Vitreous CD4^+^ and CD8^+^ cells displayed greater polyfunctional potential (TNFα^+^IFNγ^+^IL‐2^+^ and PMA/ionomycin activation) than PBMCs. Upon antigen‐specific activation *in vitro*, vitreous CD8^+^ T cells (but not CD4^+^ T cells) showed a stronger polyfunctional response than PBMCs against both MTb (in TB‐immunoreactive patients) and retinal autoantigens. Unsupervised clustering identified 15 distinct CD3^+^ T‐cell metaclusters, each with unique profiles in the vitreous and PBMCs. Significant cluster enrichment was observed among the vitreous infiltrating cells in TB‐immunoreactive cases compared to non‐TB uveitis, but no such enrichment was found among PBMCs in either patient cohort.

**Conclusion:**

The vitreous T‐cell compartment in this group of uveitis patients was functionally dominated by antigen‐responsive cytotoxic CD8^+^ T cells and was distinct from the corresponding peripheral blood compartment.

## Graphical abstract

In this article, we compare the T‐cell profiles and antigen‐specific cytokine responses between paired vitreous and peripheral blood samples from patients with sight‐threatening uveitis. The vitreous was relatively enriched in CD8 cells, exhibiting a stronger antigen‐specific polyfunctional cytokine response (against retinal and TB antigens) than the matching peripheral blood samples. Unsupervised clustering also revealed distinct profiles of CD3^+^ T‐cell metaclusters in vitreous and peripheral blood cells.

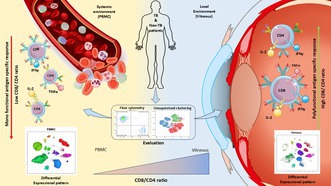

## Introduction

The intraocular immune response is modulated compared to similar responses in tissues such as the skin or gut, a phenomenon termed immune privilege. While the modulated response reduces the risk of tissue damage, it renders the eye liable to severe damage if the inflammatory stimulus subverts the immunoregulatory mechanisms.^1^, ^2^, ^3^ Consequently, intraocular inflammation (uveitis) occurs not infrequently and accounts for up to 10% of blindness and visual impairment worldwide.^4^ Uveitis is caused by infectious agents in roughly half the cases, while in many, the cause is unknown (‘undifferentiated uveitis’).^5^ Much of our knowledge of the pathogenetic mechanisms in uveitis has been derived from experimental animal models,^6^, ^7^ in which triggering antigens are inoculated either directly into the eye or into subcutaneous tissues as systemic immunogens in Complete Freund's Adjuvant. The T‐cell‐mediated (Th1 and Th17) nature of these models has been extensively described, but their predominantly acute, monophasic presentation does not adequately reflect the chronic/recurrent course of human clinical uveitis. Gaining insight into the immunological processes that underpin human uveitis might benefit from studies of biological samples from patients.

There is a long history of using blood samples to investigate immune pathogenesis in human uveitis.^8^ However, a recent study has confirmed that commonly applied blood tests (the uveitis ‘work‐up’) are relatively non‐informative for the diagnosis of uveitis.^9^ Analyses of circulating immune cells for antigen specificity have been unrewarding but the upregulation of T regulatory cells which accompanies disease remission suggests that systemic immune mechanisms may be causative in some cases.^10^ Indeed, a subset of peripheral blood CD1c^+^ dendritic cells has been implicated in the pathogenesis of non‐infectious uveitis.^11^ However, such studies are only obliquely indicative of uveitis pathogenesis, since they may not reflect mechanistic processes at the site of action, that is the intraocular tissues of the eye. In addition, since peripheral blood accounts for only 2–3% of the total T‐cell compartment,^12^ identification of the very small percentage of antigen‐specific cells that initiate disease is unlikely. A recent perspective has suggested that sampling ocular fluids may reveal more about pathogenesis and disease mechanisms in non‐infectious, undifferentiated uveitis, where the aetiology remains obscure.^13^

A significant barrier to investigating the intraocular immune response is the limited availability of tissue from the eye. Sampling uveal or retinal tissues in sufficient quantities would be detrimental to visual function. Hence, the ocular fluids—aqueous and vitreous—are increasingly used for diagnostic and immunological studies. We have previously reported antigen‐specific vitreous T‐cell responses to *Mycobacterial tuberculosis* (MTb)—early secreted antigenic target‐6 (ESAT‐6)—as well as retinal autoantigens in patients with posterior uveitis.^14^, ^15^ However, whether a correlation exists between the intraocular antigen‐specific response and circulating T‐cell responsiveness remains unknown. In the present study, we have compared antigen‐specific T‐cell cytokine responses in paired vitreous and peripheral blood samples in a cohort of patients with posterior uveitis. We find that the intraocular antigen‐specific response is primarily dominated by polyfunctional cytotoxic CD8^+^ T cells against both retinal autoantigens (in 19 vitreous samples tested) and pooled MTb peptides (in 16 vitreous samples tested), unlike in the peripheral blood. The distinctiveness of the intraocular immune response was further reinforced by differences in the unsupervised clustering of vitreous and peripheral blood T‐cell populations, which showed a dominant vitreous effector memory T‐cell population. In addition, clear differences in T‐cell clusters were observed between MTb‐positive and MTb‐negative vitreous samples but not blood samples.

## Results

### Vitreous has a higher frequency of cytotoxic CD8^+^ cells than peripheral blood, mainly of the CD45RO^+^ memory phenotypes

**Figure 1:**
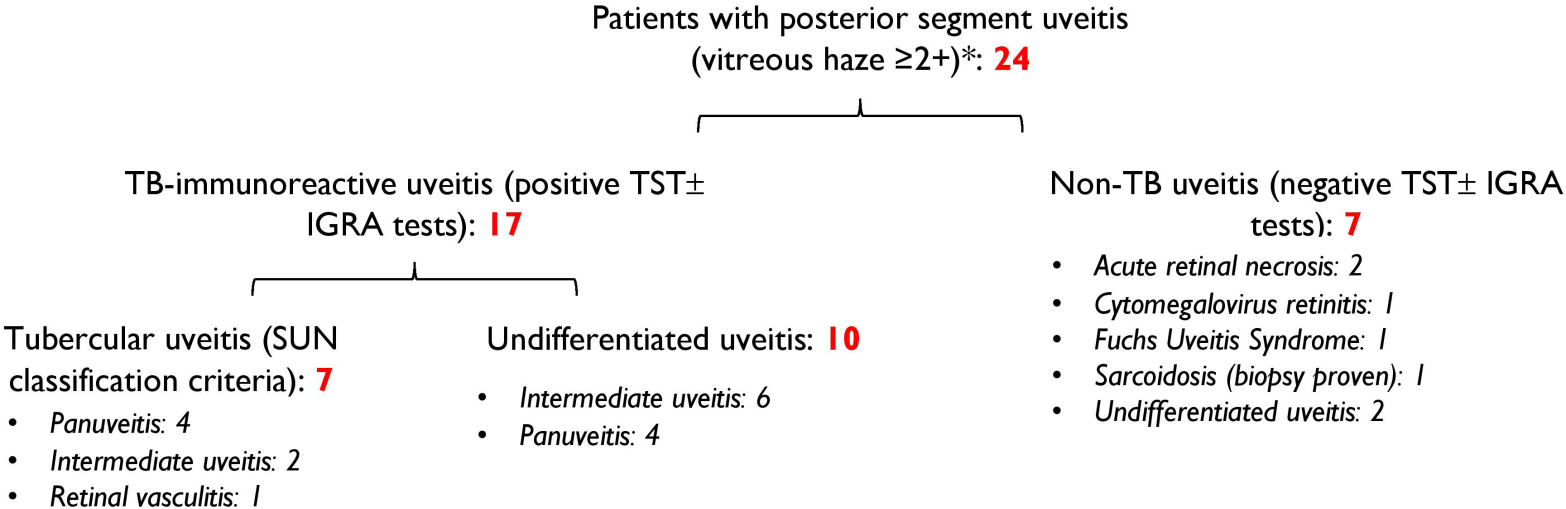
Patient demographics. A flowchart depicting patients recruited into the study and the clinical subtypes in each group.

**Figure 2:**
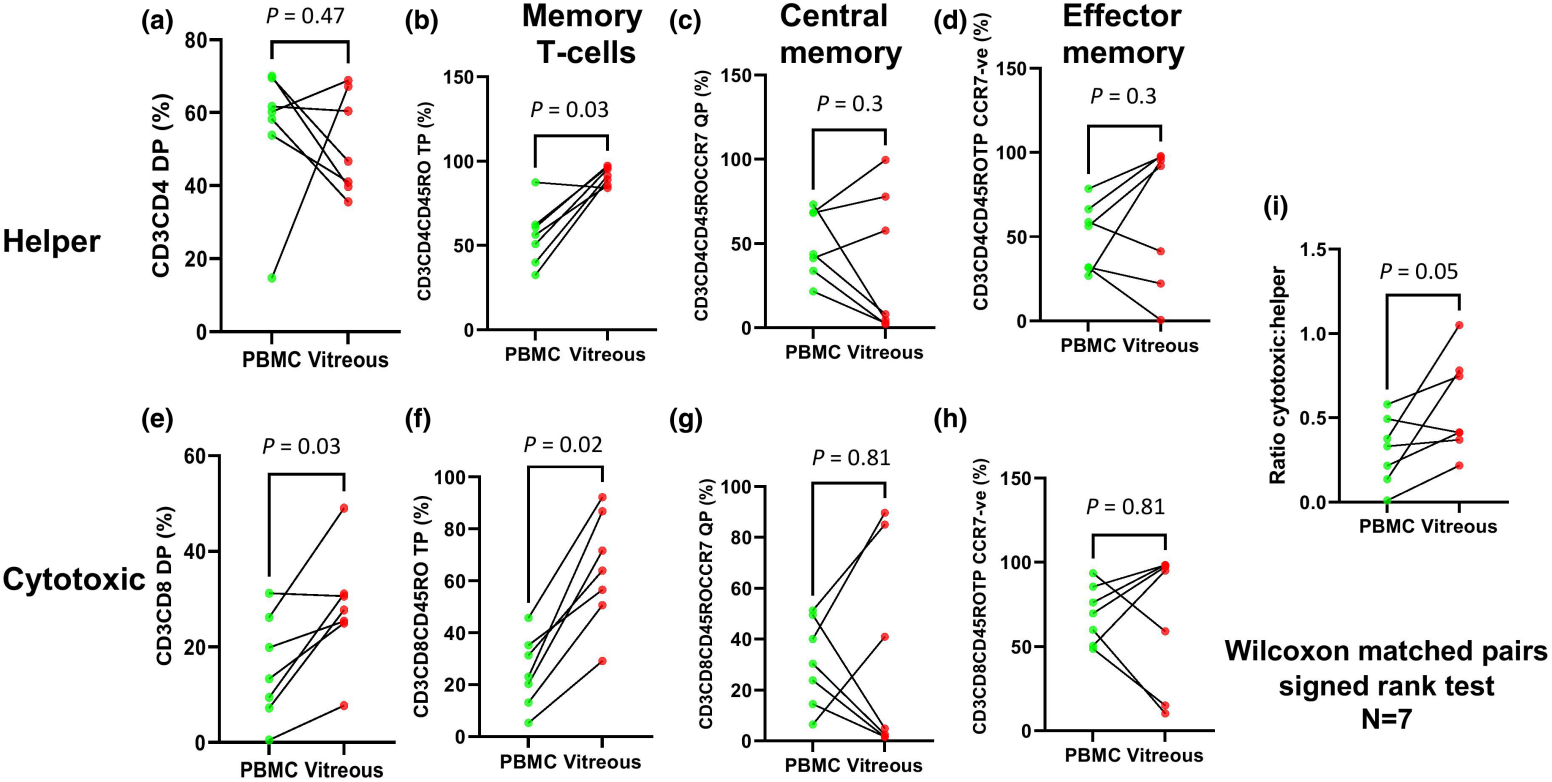
Memory T‐cell populations were expanded in the ocular compartment. Unstimulated CD3^+^ T cells from peripheral blood mononuclear cells (PBMC) and vitreous were manually gated according to the strategy outlined in Supplementary figure 2. Alterations in the proportion of T‐cell subsets between PBMC and vitreous were assessed based on surface marker expression. Comparisons of **(a)** CD3^+^CD4^+^ helper, **(b)** CD3^+^CD4^+^CD45RO^+^ memory helper, **(c)** CD3^+^CD4^+^CD45RO^+^CCR7^+^ central memory helper, **(d)** CD3^+^CD4^+^CD45RO^+^CCR7^−^ effector memory helper, **(e)** CD3^+^CD8^+^ cytotoxic, **(f)** CD3^+^CD8^+^CD45RO^+^ memory cytotoxic, **(g)** CD3^+^CD8^+^CD45RO^+^CCR7^+^ central memory cytotoxic, **(h)** CD3^+^CD8^+^CD45RO^+^CCR7^−^ effector memory cytotoxic T‐cell subsets and **(i)** the cytotoxic: helper T‐cell ratio were performed. Statistical significance was determined using the Wilcoxon rank sum test. Data represent *n* = 7 individual. DP, double positive; QP, quadruple positive; TP, triple positive.

Paired vitreous and peripheral blood samples were obtained from a total of 24 patients (Figure 1, see Methods). A representative set of paired blood and vitreous samples from this patient cohort was assessed for their content of CD3^+^ T‐cell subsets by flow cytometry using a standard gating strategy (Supplementary figure 1). Following the identification of CD4^+^ or CD8^+^ lymphocytes, sequential gating was performed to identify the state of differentiation and exhaustion (CD45RO and CCR7). Data were analysed using appropriate non‐parametric statistical tests. While absolute numbers of CD4^+^ helper and CD8^+^ cytotoxic T cells differed between blood and vitreous compartments (data not shown), the proportions of CD4^+^ T cells were comparable (Figure 2a). However, the vitreous exhibited a significantly higher percentage of CD8^+^ cytotoxic T cells than blood (Figure 2e), resulting in a higher CD8^+^ CD4^+^ ratio (Figure 2i). As expected, the frequencies of both CD4^+^ CD45RO^+^ and CD8^+^ CD45RO^+^ memory T cells were significantly greater in the vitreous (Figure 2b and f). However, no significant differences were observed in the distribution of central memory (CD45RO^+^ CCR7^+^) and effector memory (CD45RO^+^ CCR7^−^) T cells within the CD4^+^ or CD8^+^ populations (Figure 2c, d, g and h).

### Vitreous CD4^+^ and CD8^+^ T cells have a stronger polyfunctional response potential than peripheral blood cells

We assessed polyclonal T‐cell responses,^16^ in paired samples of PBMCs and vitreous infiltrating immune cells isolated from the above patient cohort by multiparametric flow cytometry. Intracellular cytokines (ICS) were assayed in cells stimulated with PMA‐ionomycin. Polyfunctionality of PBMC and vitreous infiltrating cells was compared with both conventional statistical analysis and the permutation test (the SPICE6 software). The permutation test used for this analysis asks how often a difference in numbers of polyfunctional cells between compared pies would occur simply by chance, given the samples used in the experiment. Thus, it is a more accurate approach to comparing polyfunctionality between samples than the conventional frequency analysis.

**Figure 3:**
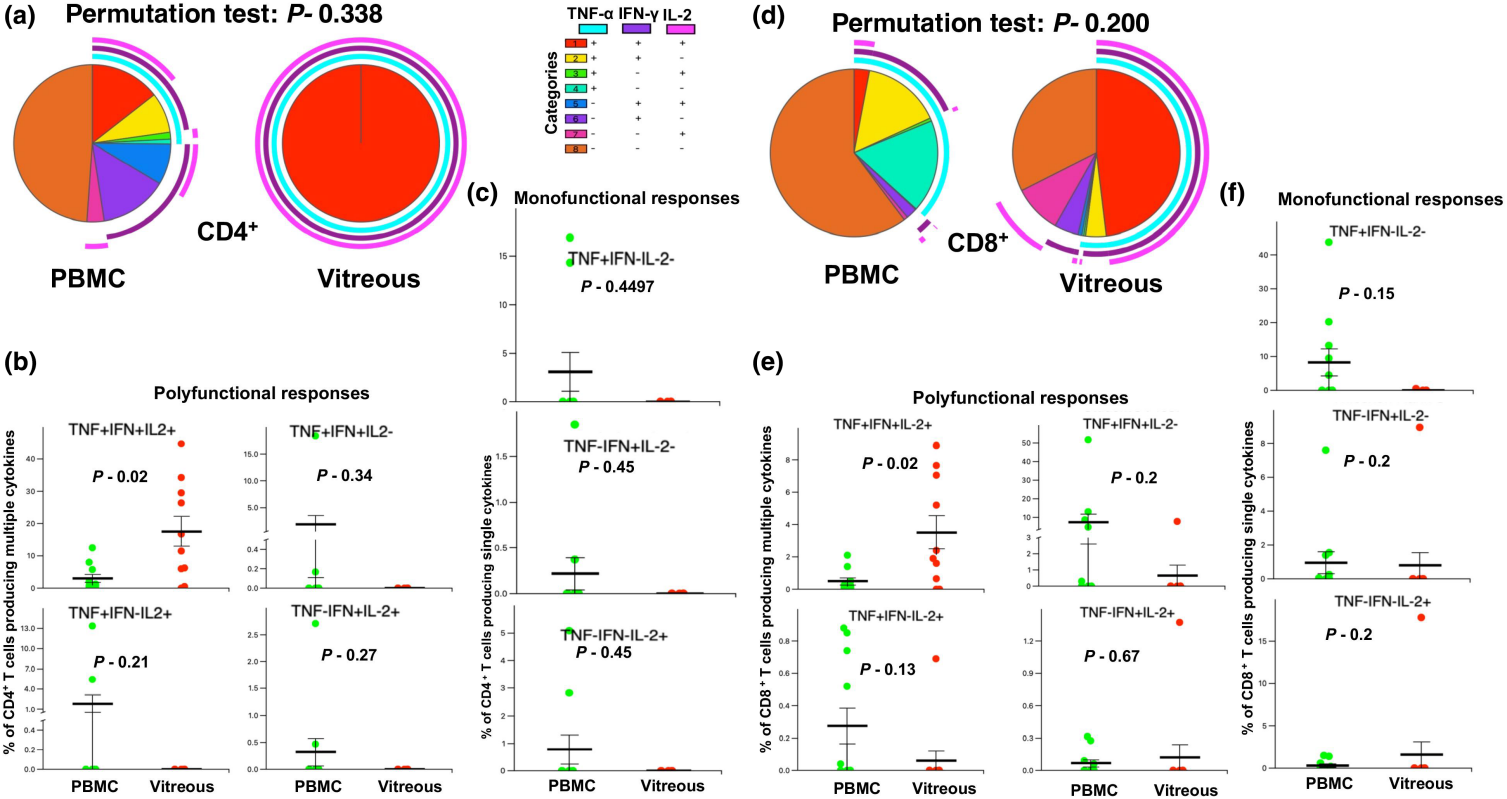
Comparison of polyfunctional and monofunctional cytokine responses of T cells stimulated with PMA/Ionomycin in paired blood and vitreous samples. Paired vitreous and peripheral blood mononuclear cells (PBMCs) were stimulated with 12.5 ng mL^−1^ phorbol 12‐myristate 13‐acetate (PMA) and 0.5 μm ionomycin (Sigma). Subsequent intracellular cytokine production was assessed and analysed using the Simplified Presentation of Incredibly Complex Evaluations 6 (SPICE 6) software. **(a)** Pie chart representing cytokine responses observed in CD4^+^ cells from blood (left) and vitreous (right). The arcs in the pie chart represent different combinations of cytokines. *In vitro* stimulation of CD4^+^ T cells isolated from the vitreous humour with PMA/ionomycin resulted in the secretion of all three cytokines tested, indicating a potential memory phenotype for these cells. **(b)** Comparison of triple‐positive and dual‐positive (TNF‐α, IFN‐γ and IL‐2) cytokines in CD4^+^ T cells between blood and vitreous. **(c)** Comparison of single‐positive cytokine in CD4^+^ T cells between blood and vitreous. **(d)** Pie chart representing cytokine responses observed in CD8^+^ cells from blood (left) and vitreous (right). The arcs in the pie chart represent different combinations of cytokines. **(e)** Comparison of triple‐positive and dual‐positive (TNF‐α, IFN‐γ and IL‐2) cytokines in CD8^+^ T cells between blood and vitreous. **(f)** Comparison of single‐positive cytokine in CD8^+^ T cells between blood and vitreous. Statistical tests were performed using the Wilcoxon rank sum test. Sample size *n* = 9.

While we did not find any statistical difference between circulating and vitreous‐infiltrating immune cells on permutation testing of different cytokine combinations, we noted differences in the cytokine expression for both CD4^+^ and CD8^+^ T cells (Figure 3a and d, respectively; Supplementary figure 2). We found a significant increase in triple‐positive (TNF‐α^+^IFN‐γ^+^IL‐2^+^) polyfunctional cells within the vitreous compartment compared to blood in the CD4^+^ and CD8^+^ T‐cell populations (Figure 3b and e). Conversely, no significant differences were detected in monofunctional (single cytokine) responses between the systemic and ocular compartments (Figure 3c and f). Interestingly, nearly 100% of the PMA‐responsive vitreous CD4^+^ cells were polyfunctional (Figure 3a), with no dual or monofunctional responses noted among them, while < 25% of the PBMC samples were polyfunctional.

### Retinal antigen‐specific autoreactive T cells are mostly restricted to the vitreous CD8^+^ T cells

**Figure 4:**
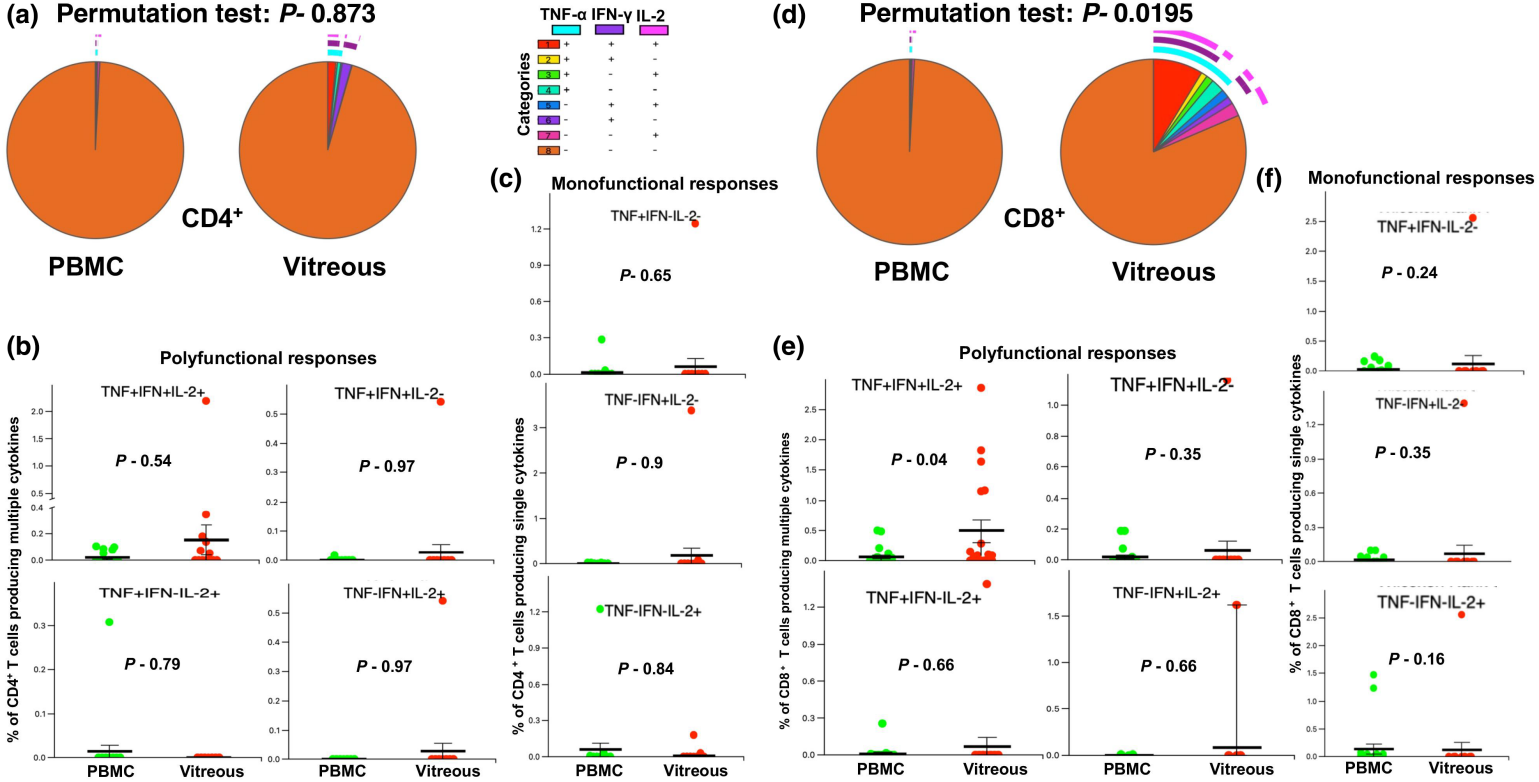
Comparison of polyfunctional and monofunctional cytokine responses of T cells stimulated with retinal antigen IRBP in paired blood and vitreous samples. Paired vitreous infiltrated and peripheral blood mononuclear cells (PBMC) were stimulated by 10 μg mL^−1^ of IRBP (1–20) peptide, a known uveitogenic T‐cell antigen, along with anti‐CD28 antibody (2 μg mL^−1^). Subsequent intracellular cytokine production was assessed and analysed using the SPICE 6 software. **(a)** Pie chart representing cytokine responses observed in CD4^+^ cells from blood (left) and vitreous (right). The arcs in the pie chart represent different combinations of cytokines. **(b)** Comparison of triple‐positive and dual‐positive (TNF‐α, IFN‐γ and IL‐2) cytokines in CD4^+^ T cells between blood and vitreous. **(c)** Comparison of single‐positive cytokine in CD4^+^ T cells between blood and vitreous. **(d)** Pie chart representing cytokine responses observed in CD8^+^ cells from blood (left) and vitreous (right). The arcs in the pie chart represent different combinations of cytokines. **(e)** Comparison of triple‐positive and dual‐positive (TNF‐α, IFN‐γ and IL‐2) cytokines in CD8^+^ T cells between blood and vitreous. **(f)** Comparison of single‐positive cytokine in CD8^+^ T cells between blood and vitreous. Statistical tests were performed using the Wilcoxon rank sum test. Sample size *n* = 19.

We have recently demonstrated autoreactive responses against retinal autoantigens in vitreous infiltrating T cells across several types of infectious and non‐infectious uveitis.^14^, ^15^ To further investigate the role of T‐cell‐mediated autoreactive responses in blood vs tissue T cells, we stimulated 19 paired PBMC and vitreous samples with the ketogenic IRBP peptide 1–20.^17^ The frequency of IRBP‐specific T‐cell clones in circulating blood was negligible among both CD4^+^ and CD8^+^ T‐cell populations, while these clones were expanded in the vitreous compartment (Figure 4a and d, respectively). Interestingly, the different permutations of cytokine co‐expression patterns exhibited a significant increase in polyfunctional cells among the vitreous CD8^+^ T cells compared to blood (Figure 4d), suggesting activation of IRBP‐specific cytotoxic T‐cell clones in the vitreous of uveitis patients.

We then analysed the differences in systemic and ocular compartments regarding polyfunctional and monofunctional immune responses to IRBP peptides. No significant differences were observed in CD4^+^ helper T‐cell cytokine responses or among CD8^+^ monocytokine and dual‐cytokine combinations (Figure 4b, c, e and f). However, the frequency of triple‐positive cytokine‐expressing CD8^+^ cytotoxic T cells was significantly higher in the vitreous compartment than in the blood (Figure 4e). We also compared the median fluorescence intensity (MFI) for each cytokine (IFN‐, TNF‐ and IL‐2) between single‐cytokine and triple‐cytokine‐producing (polyfunctional) CD8 and CD4 cells (Supplementary figure 3). As expected, the polyfunctional cells produced more cytokines per cell than the corresponding monofunctional cells.

### MTb‐antigen‐specific polycytokine responses are also predominant among vitreous CD8^+^ T cells

**Figure 5:**
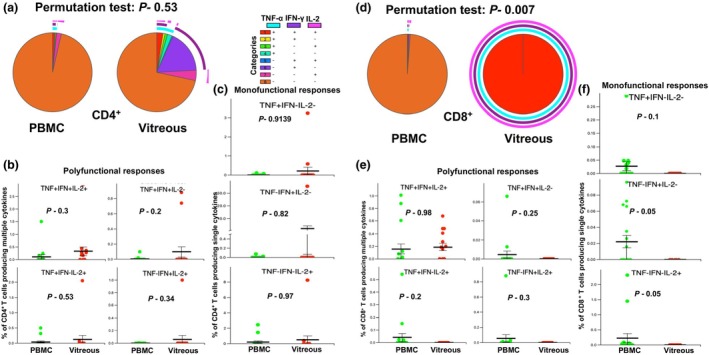
Comparison of polyfunctional and monofunctional cytokine responses of T cells stimulated with a peptide pool of *Mycobacterium tuberculosis* (MTb)‐specific ESAT‐6 and CFP10 antigens in paired blood and vitreous samples. Paired vitreous‐infiltrated and peripheral blood mononuclear cells (PBMC) were stimulated with 10 μg mL^−1^ of a peptide pool encompassing MTb antigens ESAT‐6 and CFP‐10, along with anti‐CD28 antibody (2 μg mL^−1^). Subsequent intracellular cytokine production was assessed and analysed using the SPICE 6 software. **(a)** Pie chart representing cytokine responses observed in CD4^+^ cells from blood (left) and vitreous (right). The arcs in the pie chart represent different combinations of cytokines. **(b)** Comparison of triple‐positive and dual‐positive (TNF‐α, IFN‐γ and IL‐2) cytokines in CD4^+^ T cells between blood and vitreous. **(c)** Comparison of single‐positive cytokines in CD4^+^ T cells between blood and vitreous. **(d)** Pie chart representing cytokine responses observed in CD8^+^ cells from blood (left) and vitreous (right). The arcs in the pie chart represent different combinations of cytokines. Within the vitreous humour, although the overall frequency of CD8^+^ cells recognising MTb antigens is low (less than 1%), these cells demonstrate high functional activity. Specifically, each of these MTb‐specific immune cells has the capacity to produce all three tested cytokines simultaneously. **(e)** Comparison of triple‐positive and dual‐positive (TNF‐α, IFN‐γ and IL‐2) cytokines in CD8^+^ T cells between blood and vitreous. **(f)** Comparison of single‐positive cytokines in CD8^+^ T cells between blood and vitreous. Statistical tests were performed using the Wilcoxon rank sum test. Sample size *n* = 16.

We have earlier demonstrated MTb‐antigen‐specific responses in vitreous infiltrating cells in both tubercular uveitis and undifferentiated TB‐immunoreactive uveitis.^14^, ^15^ To compare the MTb‐specific responses, we utilised a peptide pool of ESAT‐6 and CFP‐10 antigens for T‐cell activation of cell samples from 16 patients with TB‐immunoreactive uveitis.^18^ We detected a very low frequency of MTb‐specific cytokine‐secreting helper or cytotoxic T cells in PBMCs, while these cells were significantly expanded in the vitreous compartment (Figure 5a and d). As noted for IRBP‐specific responses, MTb‐specific CD8^+^ cytotoxic T cells in the vitreous also displayed high levels of activation, with nearly all the Mtb‐responsive cells positive for all three pro‐inflammatory cytokines (TNF‐α^+^, IFN‐γ^+^ and IL‐2^+^) (Figure 5d and e). Indeed, all the MTb‐responsive CD8^+^ cytotoxic T cells were polyfunctional, with no dual or monofunctional cells among them (Figure 5d, permutation test). However, this difference was not reflected when we compared the frequency of polyfunctional cells between vitreous and PBMC with conventional statistical analysis (Figure 5e, Wilcoxon rank sum test).

We observed no significant differences between blood and vitreous in CD4^+^ helper cell polyfunctional or monofunctional cytokine responses (Figure 5b and c). However, the frequency of MTb‐specific CD8^+^ cytotoxic T cells expressing single IFN‐γ or IL‐2 was significantly higher in the blood than in the vitreous (Figure 5f). Similar to IRBP, the MFI for the polyfunctional cells (producing IFN‐, TNF‐ and IL‐2 together) was greater than that of the corresponding monofunctional CD4^+^ and CD8^+^ cells (Supplementary figure 4).

### Unsupervised clustering reveals distinct T‐cell populations in vitreous and peripheral blood

**Figure 6:**
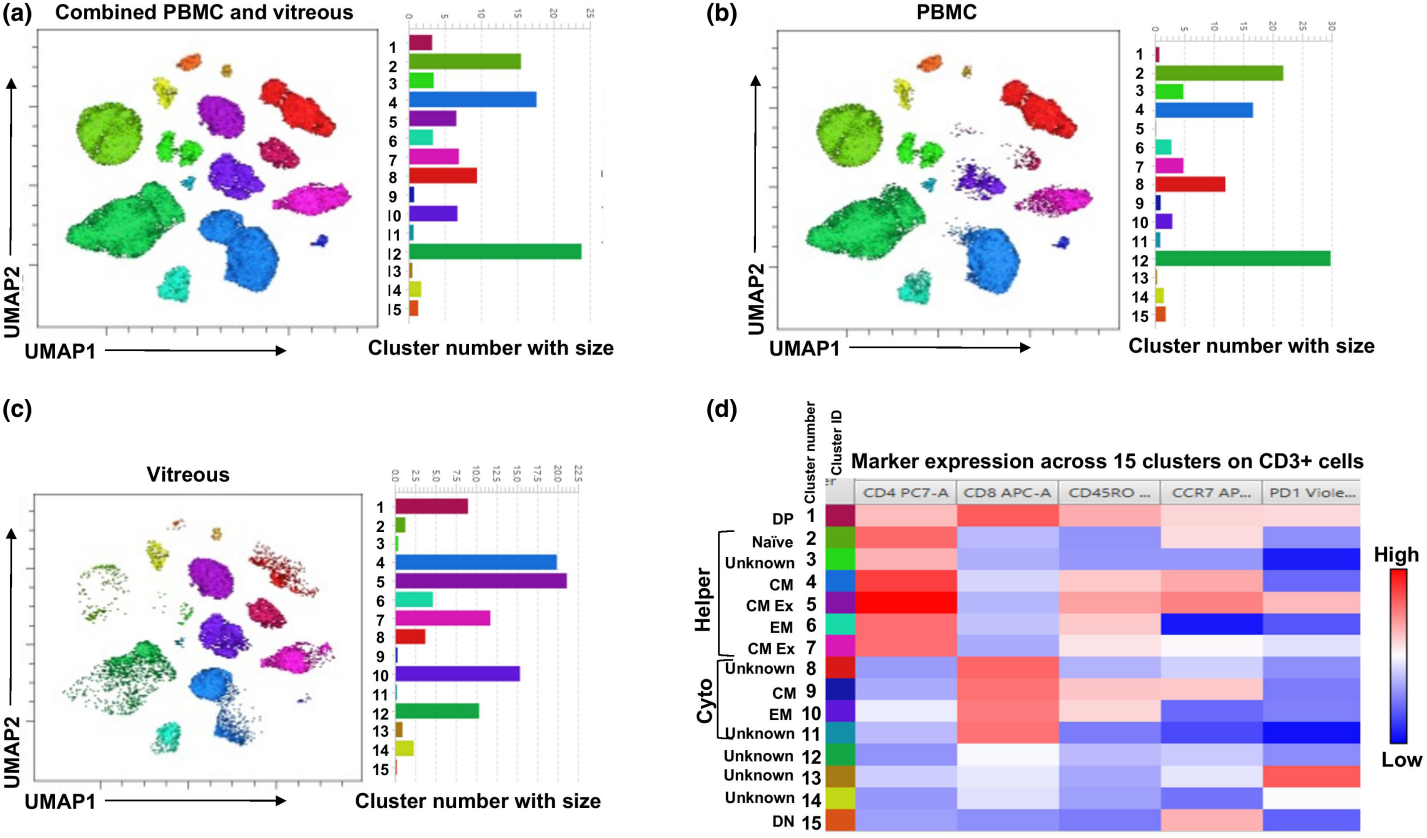
Unsupervised flow cytometry data analysis of CD3^+^ T cells from blood and vitreous and their characterisation: Manually gated 2500 CD3^+^ T‐cell events from unstimulated PBMC and vitreous samples stained with surface markers (CD4, CD8, CD45RO, CCR7 and PD‐1) were extracted and analysed by FlowSOM and visualised with uniform Manifold Approximation and Projection (UMAP). UMAP plots showing the 15 T‐cell subsets of both vitreous and PBMC combined **(a)**, only PBMC **(b)** and only vitreous **(c)**. Bar plots on the right of each UMAP plot indicate the relative sizes of clusters. **(d)** Heat map showing different CD3^+^ T‐cell clusters from PBMC and vitreous. The median fluorescent intensity (MFI) of the surface markers in each FlowSOM metacluster is represented in the heat map, with the rows showing different clusters and column markers. The colours in the heat map represent differential expressions of markers from low (blue) to high (red) calculated over cells from six PBMC and four vitreous samples.

To further characterise the T‐cell landscape in uveitis, we performed unsupervised analysis on the multiparametric flow cytometry data on a selected group of samples (six PBMC and four vitreous unstimulated samples). We obtained multidimensional information through Flow‐Self Organising Maps (FlowSOM) meta‐clustering coupled with dimensionality reduction via Uniform Manifold Approximation and Projection (UMAP).^19^ Heatmaps were then generated to visualise the differential expression of phenotypic markers. Following manual gating, we extracted and concatenated 2500 CD3^+^ T‐cell events stained with surface markers (CD4, CD8, CD45RO, CCR7 and PD‐1). The concatenated files were separated based on the sample source (PBMC or vitreous) to analyse the distribution of cell populations across clusters. Figure 6a identifies 15 distinct clusters, including six CD4^+^ helper T‐cell subsets, four CD8^+^ cytotoxic subsets, three dim CD8^+^ clusters, and one each of double‐positive (DP) and double‐negative (DN) T‐cell clusters. Figure 6b shows high‐frequency clusters in peripheral blood, including CD4^+^ helper T‐cell clusters 2 and 4 and CD8^+^ cluster 12. Conversely, the vitreous exhibited a predominance of bright CD4^+^ helper T‐cell clusters 4, 5 and 7, along with bright CD8^+^ cytotoxic cluster 10, dim CD8^+^ cluster 12 and the single DP cluster 1 (Figure 6c).

Our interpretation of the differences observed between blood and vitreous has been listed in Table 1. While clusters 5 and 7 were the largest of the clusters among the vitreous CD3^+^ cells, Cluster 13 was among the smallest. Both Clusters 5 and 7 were effectively absent among the PBMCs. Clusters 4 and 12 showed two subpopulations in the combined UMAP (Figure 6a) that were inversely represented between the blood and vitreous (Figure 6b and c). PD1 expression was noted in four clusters: one DP, two CD4^+^ (central memory, Clusters 5 and 7) and one CD8^+^ (dim CD8^+^, Cluster 13) (Figure 6d) although Cluster 13 was a relatively rare population. All four clusters were more abundant in the vitreous than in the blood.

**Table 1:** Description and relative abundance of individual clusters derived from the unsupervised flow cytometry data analysis of CD3^+^ T cells from blood and vitreous

Cluster	Phenotype	PBMC	Vitreous	Comment
1	CD4^+^CD8^+^ (double positive)	↓	↑↑	Might suggest preferential recruitment of double‐positive cells into the vitreous.
2	CD4^+^ naive	↑↑↑	↓↓	Naïve cells are expectedly more in the blood than vitreous.
3	CD4^+^ unknown	↓	↓↓↓	Non‐naïve CD4^+^ cells predominant in the blood could be regulatory T cells.
4	CD4^+^ central memory	↑↑↑	↑↑↑	Two sub‐populations are seen for this cluster in the combined UMAP—one of them stays in the blood, while other moves to the vitreous.
5	CD4^+^ central memory (exhausted)	↑	↑↑↑	Activated/exhausted cells, enriched in the vitreous likely also driving CD4 cells.
6	CD4^+^ effector memory	↓	↓	Equally present in the blood and vitreous, suggesting that these are not activated cells.
7	CD4^+^ central memory	↑	↑↑↑	CD4^+^ cells enriched in the vitreous but with low PD1, possibly representing early activated cells.
8	CD8^+^ unknown	↑↑	↓	CD8^+^ cells slightly enriched in the blood could represent non‐activated recirculating cells.
9	CD8^+^ central memory	↓↓↓	↓↓↓	Small population in both blood and vitreous.
10	CD8^+^ effector memory	↑	↑↑↑	Largest CD8^+^ cluster in the vitreous, possibly representing activated CD8^+^ cells; however, low PD1 expression suggests early activation.
11	CD8^+^ unknown	↓↓↓	↓↓↓	Small population in both blood and vitreous.
12	Unknown (? CD8^+^)	↑↑↑	↑↑↑	CD8^+^ dim populations, possibly less cytotoxic than other CD8^+^ populations; Cluster 12 is the most abundant among these, but more in the blood – possibly represents non‐activated or ‘bystander’ cells in the vitreous.
13	Unknown (? CD8^+^, exhausted)	↓↓↓	↓↓	
14	Unknown (? CD8^+^)	↓↓	↓↓	
15	CD4^−^CD8^−^ (double negative)	↓↓	↓↓↓	Small population in both blood and vitreous – could represent γδ T cells.

The following signs are used to represent a qualitative scale/relative abundance: ↓↓↓ = less than 1%, ↓↓ = 1–2.5%, ↓ = 2.5–5%, ↑ = 5–7.5%, ↑↑ = 7.5–10%, ↑↑↑ = more than 10%.

### The vitreous T‐cell landscape can distinguish TB‐immunoreactive from non‐TB‐immunoreactive uveitis

**Figure 7:**
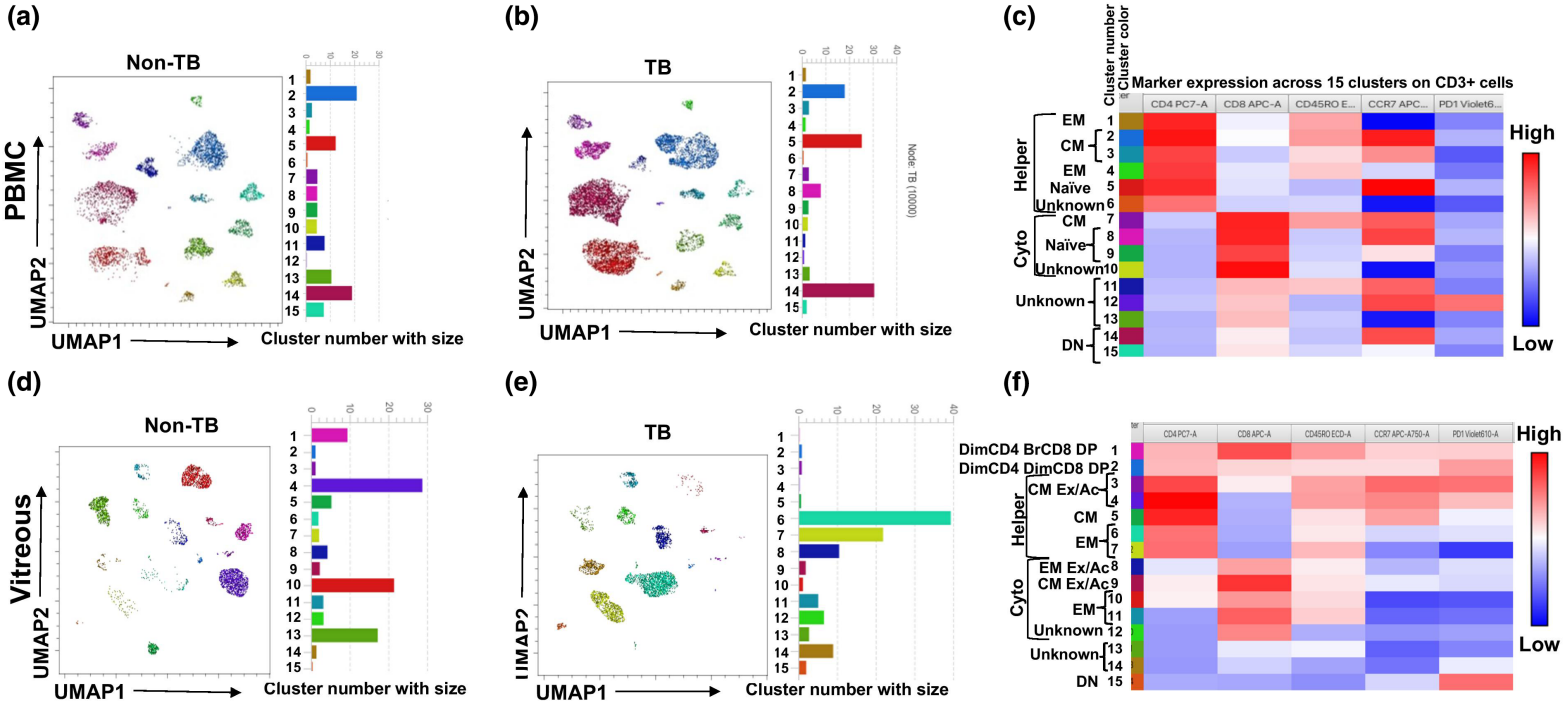
T‐cell landscape from the ocular compartment can distinguish TB‐immunoreactive from non‐TB immunoreactive uveitis: Manually gated 2500 CD3^+^ T‐cell events from unstimulated PBMC or vitreous samples stained with surface markers were categorised into two groups, non‐TB and TB, based on TST (tuberculin skin test) reactivity. PBMC samples were extracted and analysed by FlowSOM and visualised with uniform Manifold Approximation and Projection (UMAP). UMAP plots showing FlowSOM clusters from non‐TB and TB from PBMC **(a, b)** and vitreous **(d, e)**. Bar plots on the right of each UMAP plot indicate the relative sizes of clusters. Heat map showing different CD3^+^ T‐cell clusters from PBMC **(c)** and vitreous **(f)**. The median fluorescent intensity (MFI) of the surface markers in each FlowSOM metacluster is represented in the heat map, with the rows showing different clusters and column markers. The colours in the heat map represent differential expressions of markers from low (Blue) to high (red) calculated over cells of all the PBMC or vitreous samples. PBMC *n* = 6 (non‐TB = 2 and TB = 4), vitreous *n* = 4 (non‐TB = 2 and TB = 2).

A high proportion of uveitis patients show immunoreactivity to TB antigens, both in TB‐endemic and non‐endemic countries.^20^ Therefore, we wanted to investigate how the T‐cell immune profiles in peripheral blood and vitreous would be influenced by TB infections. For this, we categorised both PBMC and vitreous samples into two groups—TB (*n* = 3) and non‐TB (*n* = 2)—based on their reactivity to TST or IGRA tests. Both non‐TB samples were collected from PCR‐proven viral uveitis patients. We excluded the sarcoidosis sample (included in the Figure 6 analysis) to maintain the phenotypic distinction between TB and non‐TB samples, as described above. We extracted 2500 CD3^+^ events from vitreous and PBMC and separately concatenated them for unsupervised clustering by FlowSOM and visualisation by UMAP. The expression of different clusters in the PBMCs appeared largely similar between the TB and non‐TB groups (Figure 7a–c), apart from the appearance of a second population of central memory CD4^+^ T cells in the TB group (Figure 7b Cluster 2) and reduced expression of DN and naive T‐cell markers in samples from non‐TB patients. Clusters 2 (central memory), 5 (naive) and 14 (double negative) were enriched in both groups. However, we found clear differences between the two groups among the vitreous infiltrating T cells (Figure 7d–f). Among the CD4^+^ clusters, the non‐TB cells were enriched in a DP cluster (Cluster 1) and a central memory cluster (Cluster 4), while the TB cells were enriched in two effector memory clusters (Clusters 6 and 7). As expected, two CD8^+^ clusters (Clusters 10 and 13, both effector memory) showed substantial enrichment among the non‐TB cells, though some (Clusters 11, 12 and 14) were also mildly enriched among the TB cells. All PD1‐expressing clusters were more strongly represented among the non‐TB cells than the TB cells.

## Discussion

In this study, we have demonstrated phenotypic and functional differences in the antigen‐specific T‐cell responses between paired vitreous and peripheral blood samples during chronic intraocular inflammation. We find that the intraocular immune response is dominated by antigen‐driven polyfunctional CD8^+^ T cells, with relatively weaker contributions from the CD4^+^ cells. We also used unsupervised clustering to demonstrate that the vitreous immune cell population is not only distinct from the peripheral blood but also between two different etiological phenotypes—TB and non‐TB uveitis.

We first compared the functional attributes of the CD4^+^ and CD8^+^ T‐cell populations in the vitreous and peripheral blood. Both CD4^+^ and CD8^+^ populations in the vitreous demonstrated a significantly greater polyfunctional (TNFα^+^IFNγ^+^IL‐2^+^) potential on chemical activation (PMA/Ionomycin) than in the peripheral blood. However, the antigen‐specific polyfunctional response was largely restricted to the vitreous CD8^+^ population on activation with either retinal autoantigenic peptide (IRBP peptide 1–20) or the *Mtb* peptide pool. Surprisingly, TNFα‐ and IL‐2‐secreting monofunctional cells against *Mtb* were more numerous in the peripheral blood than in the vitreous. We suspect that these circulating monofunctional cells could be the potential precursors that acquired polyfunctionality on exposure to cognate antigens in the eye.

Our observations underscore that the effector functions of vitreous T cells are skewed towards CD8^+^ cells, even though CD4^+^ cells are numerically more abundant in the inflamed vitreous. Polyfunctionality among CD8^+^ T cells correlates with greater antimicrobial function during chronic infections and with pathogenicity during autoimmune diseases.^21^ Both conditions are driven by persistent antigenic activation and by the presence of helper CD4^+^ T cells. CD4^+^ cells upregulate expression of the co‐stimulatory molecule B7 on CD8^+^ T cells to augment their activation status and to secrete multiple cytokines, such as IL‐2, to support CD8^+^ T‐cell proliferation.^21^ That the PBMCs had a higher proportion of monofunctional antigen‐specific CD8^+^ cells against *Mtb* supports the possibility that peripherally activated monofunctional cells convert to a polyfunctional state because of persistent antigenic activation after homing to the eye, and/or because of support gained from the CD4^+^ cells in the eye.^22^ The increased cytotoxic/helper T‐cell ratio in the vitreous compared to peripheral blood further supports a heightened effector role for CD8^+^ cells in different uveitis conditions.

Antigen‐specific cytokine responses from vitreous infiltrating cells in human uveitis are rarely reported, partly because of the challenges in isolating vitreous immune cells and partly because the incriminating antigen(s) are rarely known in most uveitis conditions. We have previously demonstrated cytokine responses to mycobacterial and retinal autoantigens by vitreous T cells but were unable to distinguish between CD4^+^ and CD8^+^ responses.^14^, ^15^ Autoreactive responses among vitreous infiltrating cells have also been reported in birdshot chorioretinopathy.^23^ Unsurprisingly, we did not find a cytokine response to IRBP or *Mtb*‐peptide pool in a large proportion of the samples. This could be attributed to the low frequency of specific cytokine‐secreting cells in the overall T‐cell population responsive to the antigens/peptides being used for the activation. Application of additional surface markers of T‐cell activation such as CD69, CD40L, CD137 and OX40 (activation‐induced marker assays),^24^ could reveal a larger population of antigen‐specific T cells.

Our data on the functional characteristics of CD8^+^ T cells during chronic uveitis appear to be different from data reported from EAU studies.^25^, ^26^ In EAU, retinal CD8^+^ T cells accumulating during the persistent phase of the disease reportedly have a regulatory role and an exhausted phenotype with limited effector functions. Depletion of these cells results in the expansion of CD4^+^ T cells and CD11b^+^ myeloid cells.^25^ This contrasts with our data, where a significant proportion of CD8^+^ T cells demonstrate polyfunctional responses and have low PD1 expression. This difference could be accounted for by the lower grades of inflammation (stage of secondary regulation) at the time of sampling in the EAU studies.^27^ However, in human autoimmune disease, local CD8^+^ T cells accumulating during chronic inflammation have a metabolically active, effector phenotype driving the local inflammatory response.^28^ Single‐cell transcriptomic data of vitreous CD8^+^ T cells also reveal the expression of nearly all cytotoxic effectors in the CD8^+^ clusters during chronic uveitis. Thus, the role of local CD8^+^ T cells in chronic human uveitis might differ from that in late EAU, in which the inflammation tends to recover in the late stages.

Unsupervised clustering is now routinely used for the unbiased interrogation of both mass and classical cytometry data.^19^ We found 15 different clusters of CD3^+^ T cells that had largely complementary distribution between the vitreous and PBMC compartments. Those clusters that were enriched in the vitreous were depleted in the PBMCs, and vice versa. While manual gating revealed overall enrichment of the vitreous with CD45RO^+^ memory T cells, unsupervised clustering showed the differential distribution of the memory cell clusters between vitreous and PBMCs. While the central memory CD4^+^ population was better represented in the PBMCs (except the exhausted cluster), the effector memory CD8^+^ population was enriched in the vitreous, again underlining the prominent role of CD8^+^ cells in the effector responses. As expected, naïve T cells (CD45RO^−^CCR7^+^) were detected in the PBMC, while an unusual CD4^+^CD8^+^ double‐positive population was also enriched in the vitreous. Mature CD4^+^CD8^+^ double‐positive cells have been described in the blood and peripheral lymphoid organs (possibly because of co‐expression of transcription factors ThPOK and Runx3), but their functional role remains unclear.^29^ The greater abundance of these cells in our vitreous samples compared to blood during active inflammation suggests that they are more likely to have cytotoxic than suppressive function. Finally, all PD1‐expressing ‘exhausted’ T‐cell clusters (Clusters 1, 5, 7, 13, Figure 6d) were enriched in the vitreous. This can be explained by the high levels of cognate antigen at the site of inflammation which supports the generation of exhausted T cells.^30^ To our knowledge, this is the highest resolution of the vitreous T‐cell landscape in uveitis reported to date.

We also found complementarity between vitreous samples of TB‐immunoreactive and non‐TB uveitis. The substantial enrichment of CD8^+^ clusters among the non‐TB cells was in line with the predominance of the CD8^+^ response noted earlier in viral uveitis.^31^ The PD1^+^ exhausted T‐cell clusters were also predominant among the non‐TB samples, possibly representing higher grades of inflammation,^32^ noted previously in viral uveitis.^31^ Unlike the vitreous samples, no significant difference was found between TB and non‐TB uveitis among the PBMC samples. Nonetheless, the marked incongruity in the T‐cell landscape between the vitreous samples of the two groups and the lack of it between the corresponding PBMC samples clearly demonstrates that while T cells are activated systemically, it is not until they are recruited to, and expand in, the tissues that they drive the inflammatory process, differentially depending on their antigen specificity, for example non‐TB vs TB.

There is, however, suggestive evidence in published literature supporting the correlation between circulating immune cells and disease activity in the eye. These include the presence of activated Th1/Th17 cells in the circulation,^33^ differences in immune cell phenotypes between different disease entities (e.g. sarcoidosis and Vogt Koyanagi Harada disease), and between different stages of disease.^10^, ^11^ However, the latter two studies have reported on different cell types (regulatory T cells and myeloid dendritic cells, respectively) compared to our study. It remains unclear whether these peripheral blood alterations represent the systemic disease associated with uveitis or an immunogenetic environment that predisposes to intraocular inflammation.

Our study is limited by the relatively small number of samples and the clinical heterogeneity among those samples. Yet we found phenotypic similarities within the groups that were used for comparison, such as TB‐immunoreactive uveitis and non‐TB uveitis. The non‐TB group used for unsupervised clustering comprised samples from viral uveitis patients, thus suggesting that this type of tissue analysis may guide the clinician towards more definitive diagnosis and treatment. It also remains unknown if the vitreous infiltrating cells truly represent retinochoroidal inflammation, though cerebrospinal fluid and synovial fluid are known to be comparable to their respective tissues.^34^ The study would also have benefitted from more surface (including tissue‐resident memory T‐cell) and functional markers and a more diverse antigenic pool for T‐cell activation. We plan to incorporate these in future studies while also recruiting a larger and more homogenous group of patients. Notwithstanding the above limitations, our study provides definitive evidence of the immunological divide between tissue (vitreous) and blood immune cell communities, in addition to quantifying the heterogeneity of the vitreous infiltrating cells in high resolution. Our data will inform future studies on the immunopathogenesis of human uveitis, as well as a more accurate correlation with animal models of uveitis.

## Methods

### Patient recruitment

The study involved patients with different forms of posterior segment uveitis (infectious and non‐infectious), presenting at the LV Prasad Eye Institute, Hyderabad, a tertiary care eye centre in south India. Ethics approval for collecting vitreous and peripheral blood samples was obtained from the Institutional Review Board of LV Prasad Eye Institute (study code 2019‐133‐IM‐26). We recruited patients consenting for the study, with vitreous haze ≥ 2+ (National Institutes of Health photographic scale) at the time of surgery,^35^ and requiring diagnostic or therapeutic pars plana vitrectomy for the management of uveitis. The etiological diagnosis of uveitis was based on established clinical criteria comprising specific clinical signs, ocular imaging and laboratory investigations.^5^ PCR testing of vitreous samples was performed for specific infections when suspected but was not mandatory for the diagnosis. A total of 24 patients, 14 (58.3%) male, with a median age of 38.5 years (range 5–69 years), were included. Of these, 17 (70.8%) patients were classified as TB‐immunoreactive uveitis and seven as non‐TB uveitis (Figure 1). All patients were immune‐competent; only one had any evidence of active systemic disease (biopsy‐proven sarcoidosis). Of the TB‐immunoreactive patients, seven (41.2%) were classified as tubercular uveitis (ocular TB, based on Standardisation of Uveitis Nomenclature classification criteria),^36^ and 10 as undifferentiated uveitis. Six of the seven ocular TB patients tested polymerase chain reaction (PCR)‐positive for *Mtb*. The non‐TB uveitis (*n* = 7) comprised two patients with acute retinal necrosis (both herpes simplex virus‐1), one cytomegalovirus retinitis, one Fuchs' Uveitis Syndrome, one biopsy‐proven sarcoidosis, and two patients with undifferentiated intermediate uveitis.

### Vitreous and peripheral blood sample collection

Vitreous humour samples were obtained from uveitis patients by standardised 25‐Gauge microincision pars plana vitrectomy technique, as previously described.^37^ Briefly, following aseptic procedures, a 0.5‐mL aliquot of vitreous was initially collected for MTb PCR analysis. Subsequently, the vitrectomy procedure continued under Ringer lactate infusion, with the remaining vitreous aspirate collected into a 20‐cc syringe under controlled, slow manual aspiration. Prior to the vitrectomy surgery, peripheral venous blood samples were also withdrawn and collected in heparinised tubes.

### Isolation of immune cell populations

Vitreous samples were processed within 2 h of surgery. These were diluted 1:1 with phosphate‐buffered saline (PBS) and filtered through a 40‐μm cell strainer to remove vitreous debris. Cells were pelleted by centrifugation at 500 *g* for 20 min and washed twice with Roswell Park Memorial Institute (RPMI; Thermo Fisher Scientific, Waltham, MA, USA) 1640 medium supplemented with 10% fetal bovine serum (FBS), 100 U mL^−1^ penicillin, 100 μg mL^−1^ streptomycin and 2 mm l‐glutamine (complete RPMI 10). A small aliquot of the cell suspension was used for cell counting and viability assessment using a trypan blue dye exclusion assay on the haemocytometer. In parallel, peripheral blood mononuclear cells (PBMCs) were isolated from paired blood samples using Ficoll‐Paque PLUS (Sigma Aldrich, St. Louis, MO, USA) density gradient centrifugation.^14^ Briefly, blood was diluted with an equal volume of sterile PBS and slowly layered on the Ficoll‐Paque PLUS (Sigma) at a ratio of 2:1 in a 15‐mL Falcon tube. Samples were centrifuged at 350 *g* for 20 min at 25°C with no brake. The buffy coat was carefully aspirated and washed twice with PBS at 400 *g* for 6 min at 25°C. If present, red blood cells (RBCs) were lysed with 1× RBC lysis buffer, followed by two washes and resuspension in complete RPMI 10 media with 2% FBS.

### Antigen‐specific and polyclonal T‐cell stimulation

PBMCs and vitreous‐infiltrated immune cells were assessed for their antigen‐specific and polyclonal activation potential. For antigen‐specific stimulation, PBMCs (1 × 10^6^ mL^−1^) and vitreous cells (0.75 × 10^5^ mL^−1^) were plated in parallel on 96‐well U‐bottomed culture plates. Cells were stimulated with either 10 μg mL^−1^ of a peptide pool comprising MTb antigens ESAT‐6 and culture filtrate protein‐10 (CFP‐10; BEI Resources, NIAID, NIH, USA; Catalogue No.: NR‐50711 and NR‐50712, respectively) or 10 μg mL^−1^ of interphotoreceptor retinal binding protein peptide [IRBP, peptide 1–20 (RP20269; GenScript)], a known uveitogenic T‐cell antigen. To co‐stimulate T‐cell activation, 2 μg mL^−1^ of anti‐CD28 antibody (16‐0281‐38; Invitrogen) was added to all stimulated cultures. For polyclonal stimulation, cells were treated with 12.5 ng mL^−1^ phorbol 12‐myristate 13‐acetate (PMA) and 0.5 μm Ionomycin (Sigma). Following stimulation, cells were incubated for 14 h at 37°C and 5% CO_2_ in the presence of 10 μg mL^−1^ Brefeldin A and 2 μm monensin (added during the final 8 h) to promote intracellular cytokine accumulation.

### Flow cytometry analysis

Flow cytometry was performed to evaluate surface and intracellular markers of T‐cell activation, as described earlier, but with minor modifications. Cells were harvested and stained with LIVE/DEAD™ Fixable Blue Dead Cell Stain (L34962; Invitrogen, Thermo Fisher Scientific, Waltham, MA, USA) for 20 min to assess viability. Surface staining was performed using optimised concentrations of antibodies against CD3 (Alexa Fluor™ 700, OKT3; Invitrogen), CD4 (PE‐Cyanine7, OKT4; Invitrogen), CD8 (APC, RPA‐T8; Invitrogen), CD45RO (PE‐CF594, UCHL1; Invitrogen), CCR7 (APC‐eFluor™ 780; Invitrogen) and PD‐1 (Brilliant Violet 605™; BioLegend, San Diego, CA, USA) for 30 min at 4°C in the dark. Following washes, cells were fixed with fixation buffer (00‐5123‐43; Invitrogen) and incubated for 20 min at 4°C in the dark. Permeabilisation buffer (00‐8333‐56; Invitrogen) was added to the sample and centrifuged at 700 *g* for 6 min to permeabilise the cells. An antibody cocktail for intracellular staining (IFN‐γ PerCP‐Cyanine5.5, 4S. B3, eBioscience, Thermo Fisher Scientific; TNF‐α, eFluor 450, MAb11, eBioscience and IL‐2, Brilliant Violet 510™, MQ1‐17H12; BioLegend) was prepared using 0.5 μL of each antibody in permeabilisation buffer. The cocktail was added to the samples and incubated for 50 min at 4°C in the dark. Stained cells were washed with 2% FBS in PBS by centrifuging at 700 *g* for 6 min. The supernatant was discarded, and cells were resuspended in PBS with 2% FBS (Thermo Fisher Scientific). Samples were acquired on CytoFLEX S N2‐V3‐B5‐R3 flow cytometer (Beckman Coulter, Brea, CA, USA). Data analysis was performed using the FlowJo (v.10.10) (BD Bioscience, Ashland, OR, USA) and SPICE 6 software (https://niaid.github.io/spice). For intracellular cytokine staining gating, we followed the guidelines established by the International Multicenter Proficiency Panel conducted by the Cancer Immunotherapy Consortium.^38^, ^39^ We employed fluorescence‐minus‐one (FMO) controls as the primary method to establish accurate positivity thresholds for delineating cytokine‐positive and cytokine‐negative populations (Supplementary figure 5). While cytokines with low expression may occasionally exhibit subtle overlap, FMO controls allow rigorous discrimination by accounting for spectral spillover and background fluorescence.

### Unsupervised clustering and dimensionality reduction

PBMC and vitreous samples were overnight cultured in complete RPMI 10 media. These samples were stained with surface markers and acquired in CytoFLEX S N2‐V3‐B5‐R3 (Beckman Coulter) and compensated by the CytExpert software (Beckman Coulter) as described in the above section. Flow cytometry FCS 3 files from these unstimulated PBMC and vitreous samples were imported into the FlowJo software (version 10.10) and manually gated to remove debris, non‐lymphocytes, doublets and dead cells (Supplementary figure 1). To ensure equal representation of peripheral blood and vitreous infiltrated lymphocytes for unsupervised analysis, we exported 2500 CD3^+^ events from each sample. These files were concatenated and clustered into 15 metaclusters by a self‐organising map‐based FlowSOM algorithm. Subsequently, dimensional reduction was done by the Uniform Manifold Approximation and Projection (UMAP) through the UMAP plugin.^40^ FlowSOM metaclusters were overlaid on the UMAP plots using the built‐in ClusterExplorer of FlowJO.

### Statistical analyses

The Wilcoxon matched‐pairs signed‐rank test and Mann–Whitney *U*‐test were employed to compare median values between paired and independent groups, respectively. Statistical analyses were performed using Prism (version 9.0; GraphPad) and SPICE 6 (ImmunoTechnology Section, VRC/NIAID/NIH).

## Conflict of interest

The authors declare no conflict of interest.

## Author contributions

**Kaiser Alam:** Conceptualization; data curation; formal analysis; investigation; writing – original draft. **Arun Raina:** Investigation; methodology; visualization. **Bibhuprasad Das:** Investigation; methodology. **Sandhya Bhanja:** Formal analysis; investigation; methodology; visualization. **Sayantan Ghosh:** Investigation; methodology; visualization; writing – review and editing. **John V Forrester**: Conceptualization, Formal Analysis, Visualisation, Writing – Revision and Editing. **Soumyava Basu**: Conceptualization, Formal Analysis, Funding Acquisition, Methodology, Investigation, Project Administration, Visualization, Writing – Original Draft Preparation, Writing – Revision and Editing.

## Supporting information


Supplementary figures 1–5

## Data Availability

The data that support the findings of this study are available on request from the corresponding author. The data are not publicly available due to privacy or ethical restrictions.
