# Supplementary material for: Antigen‐specific polyfunctional cytotoxic T cells differentiate intraocular from peripheral blood immune responses in posterior uveitis
Source: Clin Transl Immunology. 2025 May 15;14(5):e70036. doi: 10.1002/cti2.70036 (PMC12081828; doi:10.1002/cti2.70036)

Supplementary figure 1: Gating strategy for antigen-specific cytokine responses

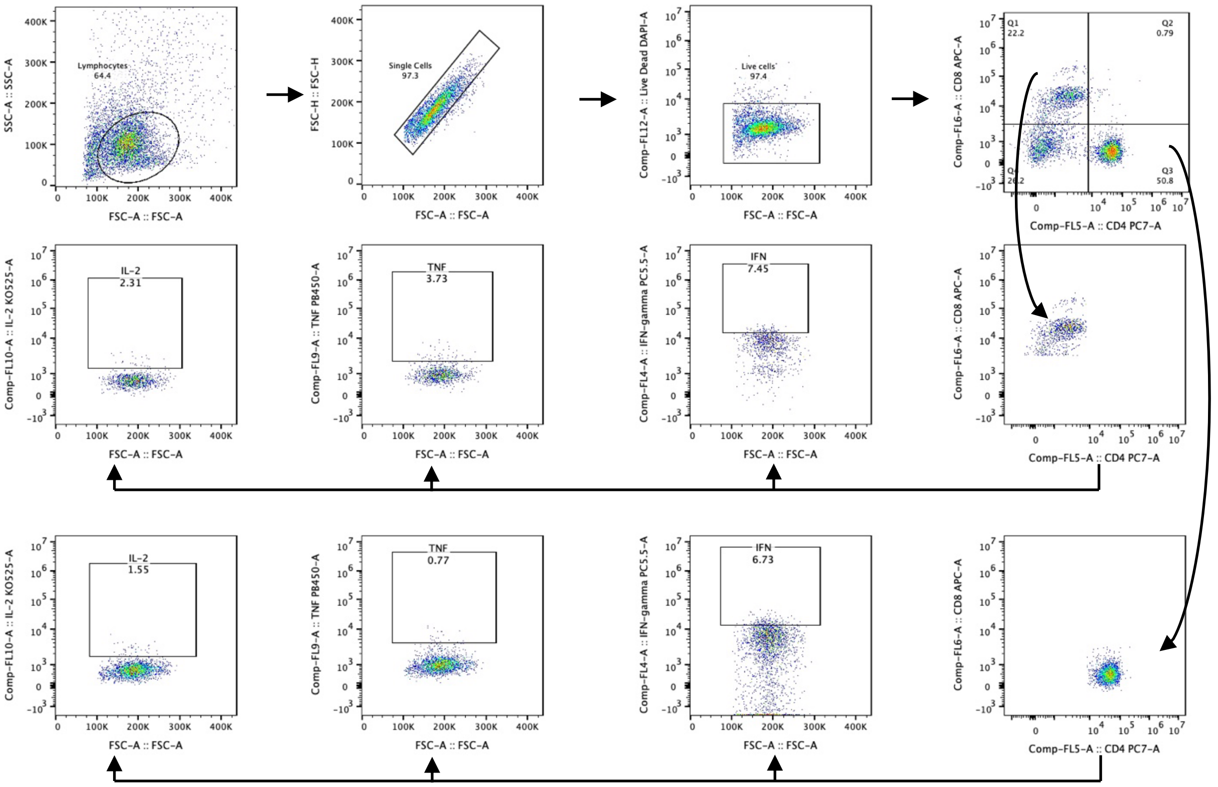

Supplementary figure 2: Gating strategy for phenotype analysis of CD3+ cells

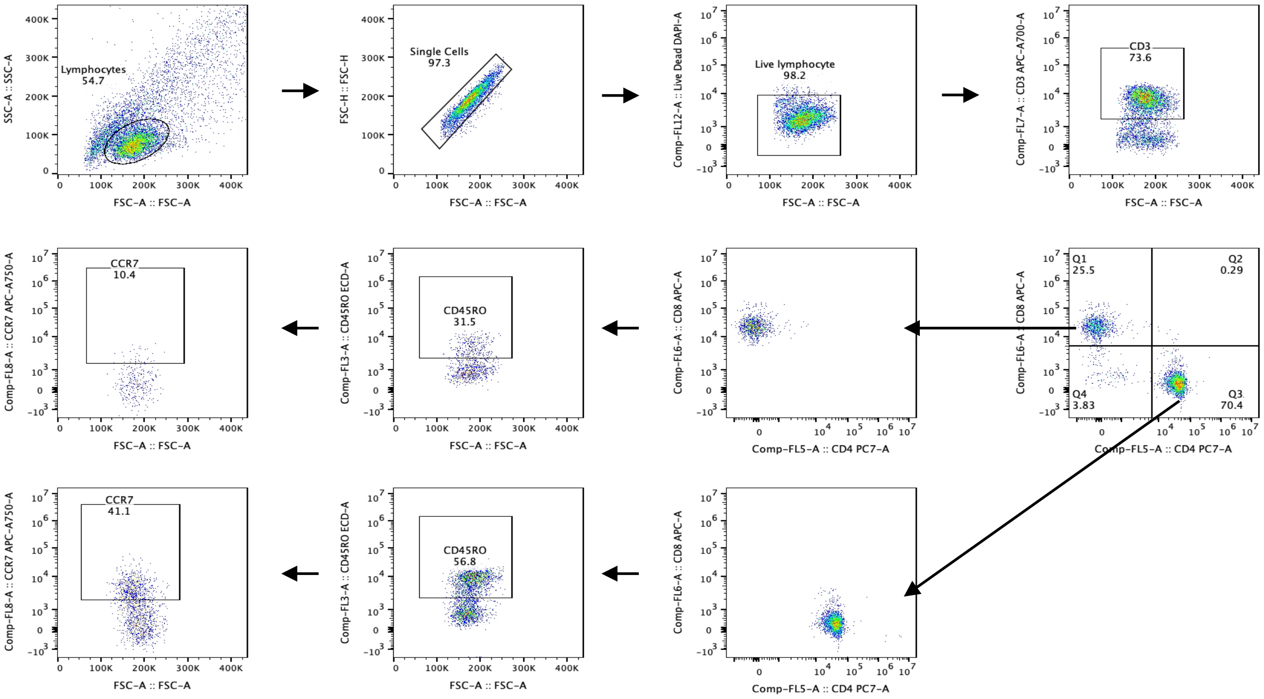

<u>Supplementary figure 3</u>: Comparison of monofunctional and polyfunctional CD8⁺ T cell responses in the vitreous fluid of posterior uveitis patients. CD8⁺ T cells were isolated from vitreous fluid and stimulated *in vitro* with either (a) retinal autoantigen interphotoreceptor retinoid-binding protein (upper panel) or (b) *Mycobacterium tuberculosis* specific peptide pool containing ESAT6 and CFP10 (lower panel). Intracellular cytokine staining for IFN-γ, TNF-α, and IL-2 was performed, and cytokine production was quantified by flow cytometry. The median fluorescence intensity (MFI) of each cytokine was compared between CD8⁺ T cells exhibiting single positivity for IFN-γ (IFN-γ 1+), TNF-α (TNF-α 1+), and IL-2 (IL-2 1+) and those exhibiting triple positivity (3+) for all three cytokines. Statistical tests were calculated using the Wilcoxon matched pairs signed rank test. The sample size for (A) N=13 and (B) N=10.

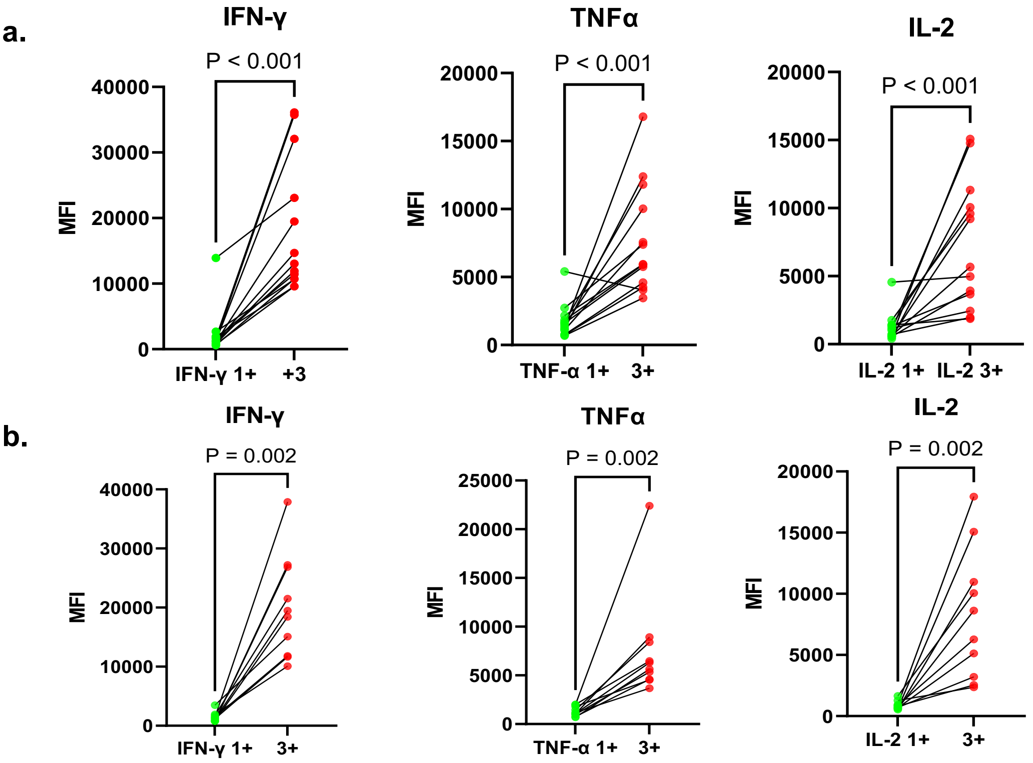

<u>Supplementary figure 4</u>: Comparison of monofunctional and polyfunctional $CD4^+$ T cell responses in the vitreous fluid of patients with posterior uveitis. $CD4^+$ T cells were isolated from vitreous fluid and stimulated *in vitro* with either (a) retinal autoantigen interphotoreceptor retinoid-binding protein (upper panel) or (b) a *Mycobacterium tuberculosis* specific peptide pool containing ESAT6 and CFP10 (lower panel). Intracellular cytokine staining for IFN-γ, TNF-α, and IL-2 was performed, and cytokine production was quantified by flow cytometry. The median fluorescence intensity (MFI) of each cytokine was compared between $CD4^+$ T cells showing single positivity for IFN-γ (IFN-γ 1+), IL-2 (IL-2 1+), or TNF-α (TNF-α 1+) and those showing triple positivity (3+) for all three cytokines. Statistical tests were calculated using the Wilcoxon matched pairs signed-rank test. The sample size for (a) is N=7, 6, and 6, respectively, for IFN-γ, TNF-α, and IL-2, and for (b) is N=7, 8, and 8, respectively, for IFN-γ, TNF-α, and IL-2.

a.

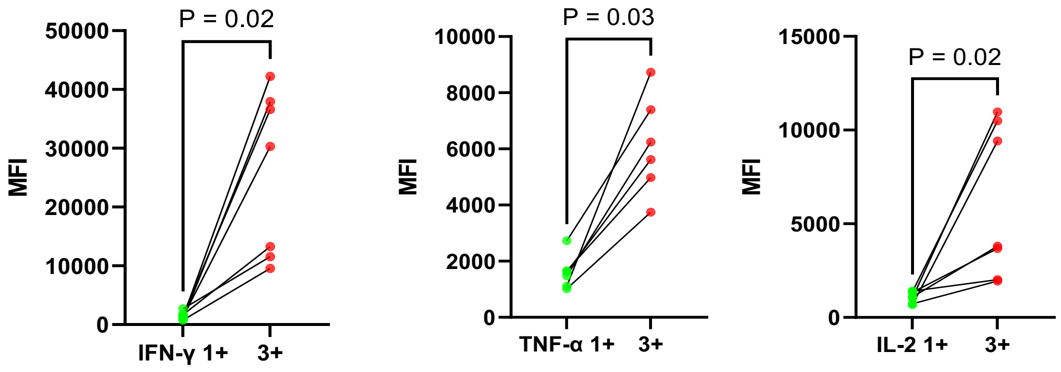

b.

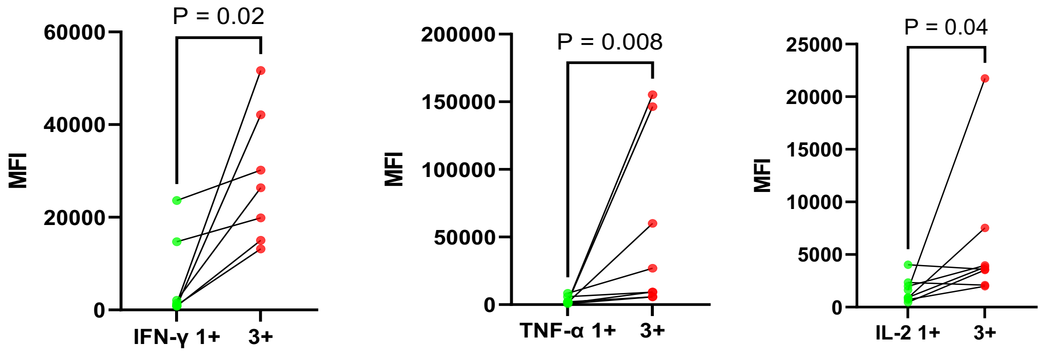

Supplementary figure 5: Gating specificity of markers determined by Fluorescence-minus-one (FMO) control

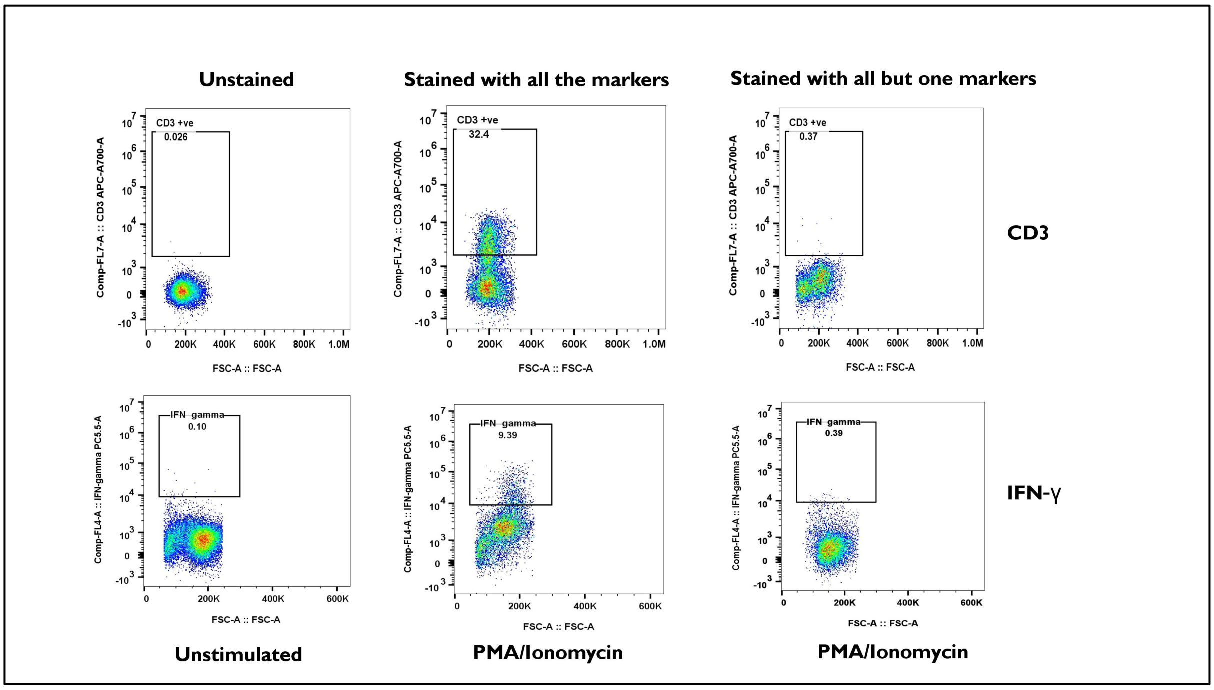

Supplement: Supplementary file 1 — Supplementary figures 1–5 [file CTI2-14-e70036-s001.pdf]
